# Preparation of 5′-O-(1-Thiotriphosphate)-Modified Oligonucleotides Using Polymerase-Endonuclease Amplification Reaction (PEAR)

**DOI:** 10.1371/journal.pone.0067558

**Published:** 2013-07-04

**Authors:** Biao Li, Shihua Dong, Jiajun Wu, Jianye Zhang, Gang Chen, Quanjiang Dong, Xinhong Zhu, Xiaolong Wang

**Affiliations:** 1 Department of Biotechnology, Ocean University of China, Qingdao, Shandong, People's Republic of China; 2 Qingdao Municipal Hospital, Qingdao, Shandong, People's Republic of China; University of Helsinki, Finland

## Abstract

Antisense oligonucleotides (ASODNs) have been widely used as an important tool for regulating gene expression, and developed into therapeutics. Natural ODNs are susceptible to nuclease degradation, nucleic acid analogues, however, have less side effects, stronger stability and more potent activities. Large-scale *de novo* synthesis of a certain oligonucleotide has been very difficult and costly. In a previous preliminary study, we developed the polymerase-endonuclease amplification reaction (PEAR) for amplification and large-scale preparation of natural antisense ODNs. Here we extended the method in preparation of a widely used modified oligonucleotide with 5′-O-(1-Thiotriphosphate) modifications. Using electrospray ionization liquid chromatography mass spectrometry (ESI/LC/MS) detection, the purity of the PEAR product was measured as high as 100.0%. Using PEAR a large amount of a specific oligonucleotide can be produced starting from a small amount of synthetic seeds. It is suggested that PEAR can be a useful tool for large-scale production of modified oligonucleotides.

## Introduction

Synthetic oligonucleotides (ODNs) have been widely used as an important tool for regulating gene expression, and developed into drugs for gene therapy, especially antisense oligonucleotides (ASODNs) [1] and CpG oligonucleotides (CpG-ODNs) [2]. Usually, ASODNs are used to inhibit the expression of pathogenic or viral genes by targeting their transcripts, including messenger RNA (mRNA) or microRNA (miRNA). Recently, however, Modarresi and his colleagues reported that inhibition of a natural antisense transcript (NAT), BDNF-AS, by ASODNs or siRNAs can transiently and reversibly upregulate the expression of a specific gene, brain-derived neurotrophic factor (BDNF), leads to increased protein levels and induces neuronal outgrowth and differentiation both *in vitro* and *in vivo* [3]. Because ASODNs are in principle simpler and more convenient than siRNAs, and due to their drug-like properties, their ability to specifically regulate gene expression, both down and up, holds great therapeutic promise for them. Fomivirsen (Vitravene) [4] for cytomegalovirus retinitis and Mipomersen (KYNAMRO) [5] for severe hypercholesterolemia are by far the only two oligonucleotide drugs that has been approved for marketing, however, dozens of ASODNs are undergoing phase I/II clinic trials [6]–[8].

Natural ODNs are known to be susceptible to nuclease degradation *in vivo* and sometimes has serious off-target side effects [9], nucleic acid analogues incorporated with one or more appropriate chemical groups, however, were shown to have less side effects, stronger stability and more potent activities than their corresponding natural counterparts. Hence, in the past two decades numerous studies on modified ODNs have been reported for applications related to target-validation or therapeutic studies. For an example, as early as in 1998, Persico and his colleagues injected 1.7 nmoles of anti c-fos oligonucleotides into medial prefrontal cortical, revealed a ca. 3 h half-life for phosphothioate and a ca. 15 min half-life for phosphodiester oligonucleotides [10]. More recently, in 2010, Lanford treated chronically infected chimpanzees with a locked nucleic acid (LNA) modified oligonucleotide (SPC3649) complementary to miRNA miR-122 leads to long-lasting suppression of hepatitis C virus (HCV) viremia, with no evidence of viral resistance or side effects in the treated animals [11].

At present, oligonucleotides are produced mostly by chemical synthesis. Large-scale synthesis of a certain oligonucleotide by *de novo* synthesis has been very difficult and costly, since it requires expensive equipment, hazardous chemicals and tedious purification process. In addition, synthetic ODNs are often contaminated with highly homologous failure sequences. A few studies have been reported that modified nucleic acid analogues were produced by enzymatic reactions, such as primer extension [12], polymerase chain reaction (PCR) [13], in vitro transcription [14], and nicking enzyme amplification reaction [15]. However, these methods might not be suited for large-scale production of oligonucleotides, since the yields of reactions are largely limited by the concentrations of templates and primers added. On the other hand, isothermal reactions such as exponential amplification reactions (EXPAR) [16] and rolling circle replication (RCR) [17]–[18] have been developed for amplifying oligonucleotides, but they have not been validated for preparing oligonucleotides with modified groups. Previously, we developed a new thermal cyclic reaction, polymerase-endonuclease amplification reaction (PEAR) [19], and demonstrated the use of it for large-scale enzymatic production of a natural ASODN. Here, we report the extension of the PEAR method for preparing of oligonucleotides incorporating 5′-O-(1-Thiotriphosphate) modifications.

## Results

### PEAR Amplification of PS Modified Oligonucleotide

**Figure 1:**
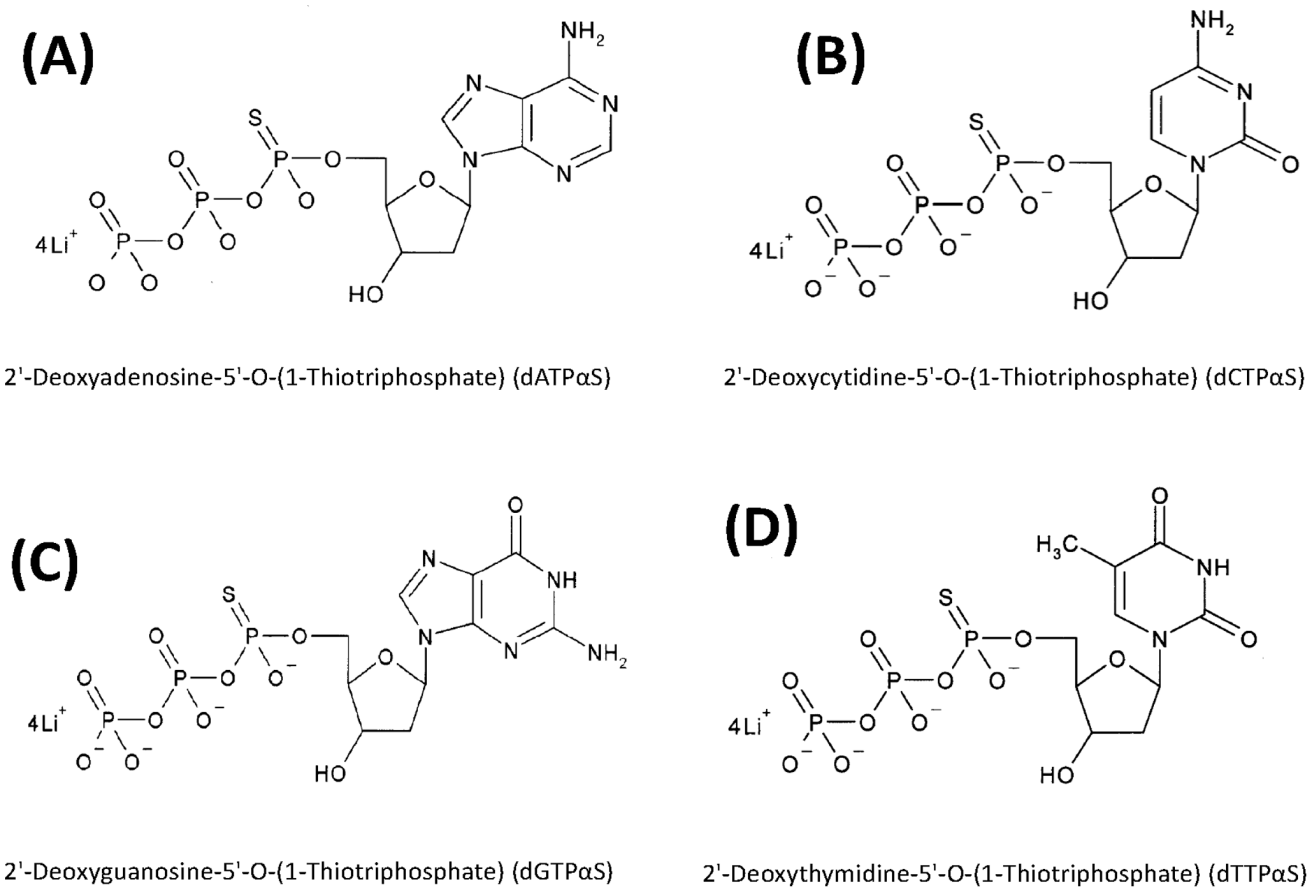
Molecular structure representation of dNTPαSs.

**Figure 2:**
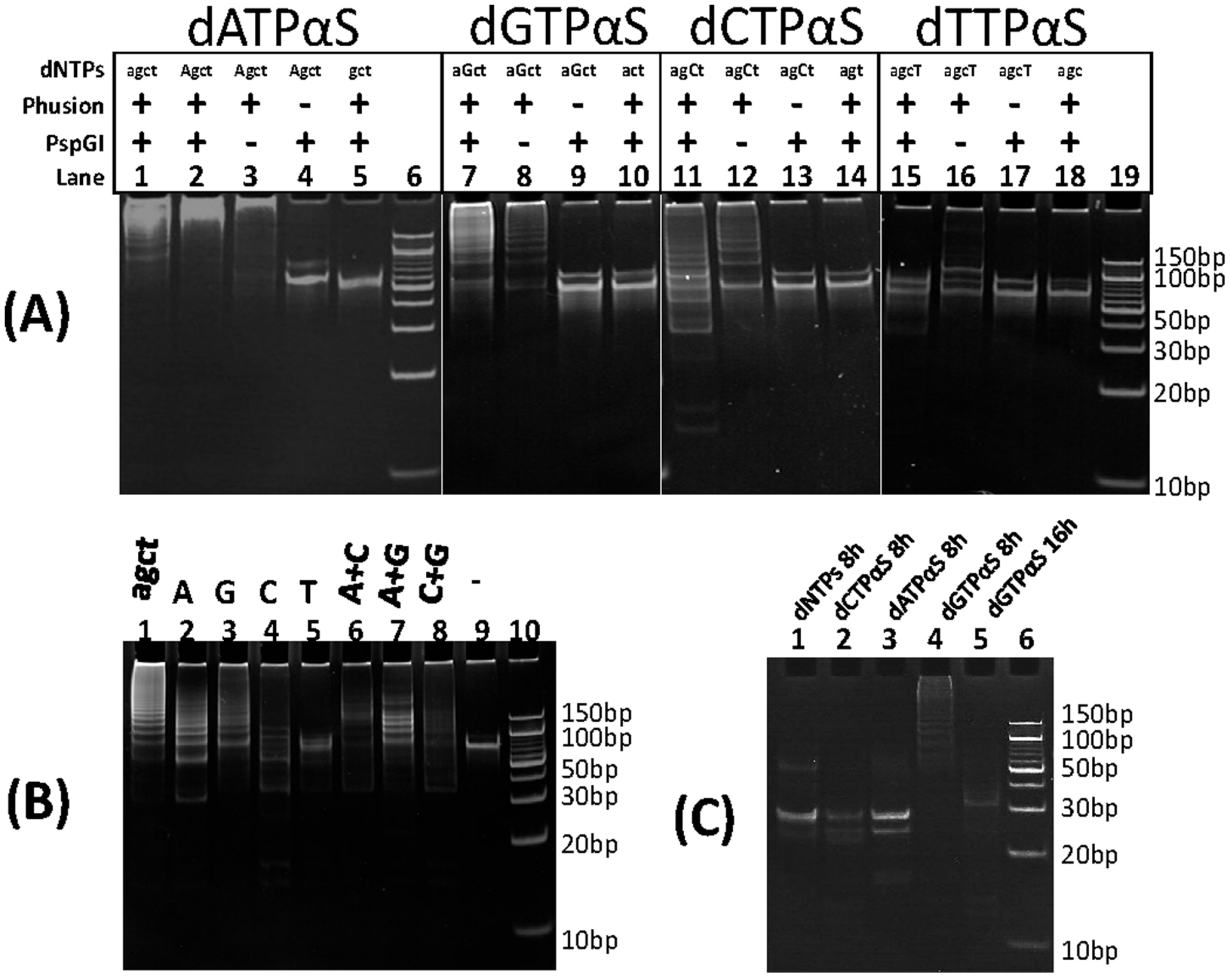
PAGE electrophoresis of PEAR products. For dNTPs, lowercase letters (agct) represents natural dNTPs, and uppercase letters (AGCT) represents dNTPαSs. (A) PEAR products incorporating natural or dATPαS, dGTPαS, dCTPαS, dTTPαS: Lane 1: natural dNTPs; Lane 2: dATPαSs; Lane 3: No PspGI control; Lane 4: No Phusion DNA polymerase control; Lane 5: No dATP control; Lane 6∶10bp DNA ladder; Lane 7: dGTPαS; Lane 8: No PspGI control; Lane 9: No Phusion DNA polymerase control; Lane 10: No dCTP control; Lane 11: dCTPαSs; Lane 12: No PspGI control; Lane 13: No Phusion DNA polymerase control; Lane 14: No dCTP control; Lane 15: dTTPαSs; Lane 16: No PspGI control; Lane 17: No Phusion DNA polymerase control; Lane 18: No dTTP control; Lane 19∶10bp DNA ladder. (B) PEAR products incorporating one or two kind of dNTPαSs: Lane 1: natural dNTPs; Lane 2–5: one kind of dNTPαSs; Lane 6–8: two kind of dNTPαSs; Lane 9: No dNTPs control; Lane 10∶10bp DNA ladder; (C) Full digestion of PEAR products incorporating different dNTPs or dNTPαSs.

We conducted PEAR reactions by using one or two dNTPαSs (dATPαS, dGTPαS, dCTPαS or dTTPαS) (Figure 1), to substitute their corresponding natural dNTPs. As shown in Figure 2A, a series of DNA bands representing tandem repeats of the target were seen in the PAGE electrophoresis of a natural PEAR product amplified using only unmodified dNTPs (lane 1). The PEAR reaction was shut down completely when one of the four essential substrates, dATP, was absent (lane 5), and products could be readily detected if dATPαS was added into the reaction (lane 2), thus indicating efficient incorporation of dATPαS into PEAR products. The yield of PEAR products depends primarily on the number of thermocycles. The ultimate maximum yield of modified PEAR products, ca. 200 ng/µL, is basically equivalent to that of the natural ones, which is not limited by the initial concentration of the target and the template, but by the concentration of substrates (dNTPs or dNTPαSs).

As described previously [19], PEAR requires a thermostable DNA polymerase (Taq) and a thermostable endonuclease (PspGI), and relies on the “*slipping and cleaving mechanism*”. An exponential PEAR consist a slipping reaction that extends the number of repeats in the PEAR product increases linearly, and a subsequent cleaving reaction that drives the number of molecules increases exponentially. When a thermostable DNA polymerase was present, but PspGI was absent (Figure 2A, lane 3), the slipping reaction was proceeding while the cleaving reaction ceased. Therefore, the products were extended but not cleaved, so that during the PEAR reaction only the number of repeats increased, but the number of molecules did not. In such a linear PEAR, the reaction rate is much slower, but the lengths of the products are longer, than that of the exponential PEAR.

For the other dNTPαSs, Phusion DNA polymerase incorporated both dGTPαS (Figure 2A, lane 7) and dCTPαS (lane 11), but not dTTPαS (lane 15), into PEAR products. The exponential PEAR was completely abolished when dTTP was replaced with dTTPαS, while the linear PEAR produced a ca. 100-bp band and some faint upper bands (lane 16). It seems that Phusion DNA polymerase accepted dTTPαS as its substrate, but only at a very slow rate. When PspGI was present, all of the extended products were cleaved, so that no duplex repeats could be produced, and thus the exponential amplification could not take place.

It has been reported in the attempted incorporation of a contiguous segment of locked nucleic acid (LNA) nucleotides by primer extension, Phusion DNA polymerase was able to incorporate up to three, eight and five LNA nucleotides successively, respectively for LNA-A, LNA-T and mixed LNA-A+LNA-T nucleotides [12]. It was assumed that DNA polymerase extension was interrupted when it encountered too many successive modified bases when two kind of modified nucleotides were added into a primer extension or PCR reaction. However, here we observed that successive incorporation of a contiguous segment of thiotriphosphate nucleotides is allowed in PEAR reaction. As shown in Figure 2B, PEAR reactions using each of the three combinations of two dNTPαSs, including dATPαS+dGTPαS, dGTPαS+dCTPαS, and dATPαS+dCTPαS, were all successful. In addition, as shown in Figure 2C and Figure S1 in File S1, modified PEAR product incorporating dGTPαS are much more resistant to endonuclease digestion than the natural products: the natural product and PEAR products incorporating dATPαS or dCTPαS were fully digested by PspGI in 4 or 8 h, the product incorporating dGTPαS, however, were not fully digested in 8 h, but in16 h.

### LC/MS Profiling of PEAR Amplified Oligonucleotides

**Figure 3:**
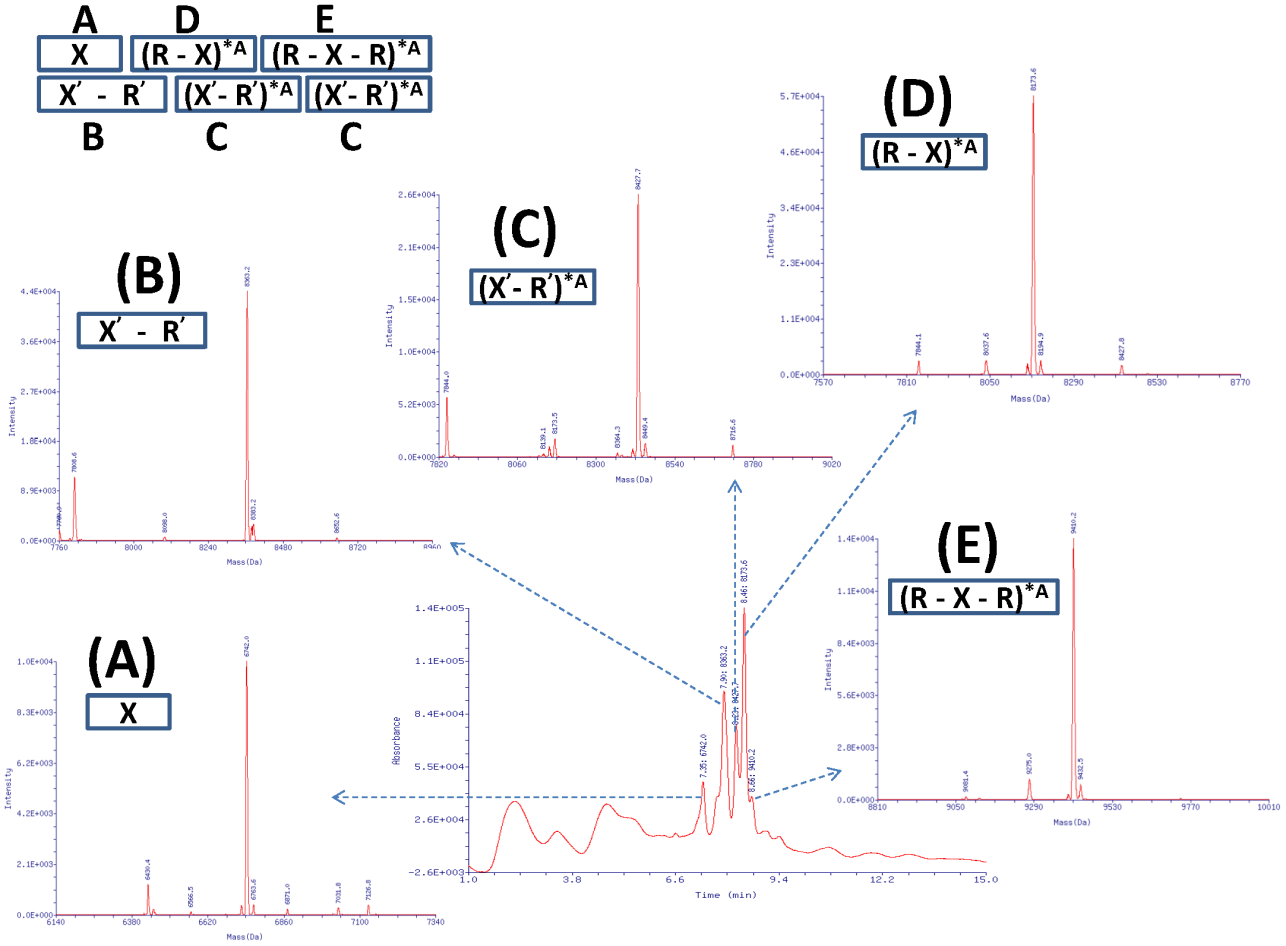
The LC/UV Chromatogram and Deconvoluted Mass Spectrum of the *A PEAR product. Components: (A) RT = 7.35 min: MW = 6742.0; (B) RT = 7.90 min: MW = 8363.2; (C) RT = 8.23 min: MW = 8427.7; (D) RT = 8.46 min: MW = 8173.6; (E) RT = 8.66 min: MW = 9410.2; See Table S1 in File S1 for detailed characterization of components.

We employed a well-established LC/MS technique [20] to measure the molecular weight (MW), confirm the molecular structure and profile the components of the PEAR products. As shown in Figure 3 and Table S1 in File S1, in the digestion of the PEAR product incorporating dATPαS (*A), there are five ODN strands, including a pair of unmodified strands which is the original seeds (target and probe), and three modified strands that were the product of PEAR amplification.

Among the five ODN strands, A, B and C are all in full length, while the other two ones, D and E, were truncated by one or two 3′-terminal bases. The sequencing results shows that in the PEAR products most of the repeats were perfect, therefore, the truncations of the 3′-terminal Gs were not caused by incomplete reaction to extend the 3′-terminus to full length, but occurred after the products had been cleaved into monomers. Because truncations happened only in modified strands, but not in unmodified strands, they must be caused by the 3′ to 5′ exonuclease activity of the Phusion High Fidelity DNA polymerase, which was triggered by the modified bases. However, the truncations were ceased when a modified base was encountered, owing to thio-modified bases are resistant to exonuclease.

**Figure 4:**
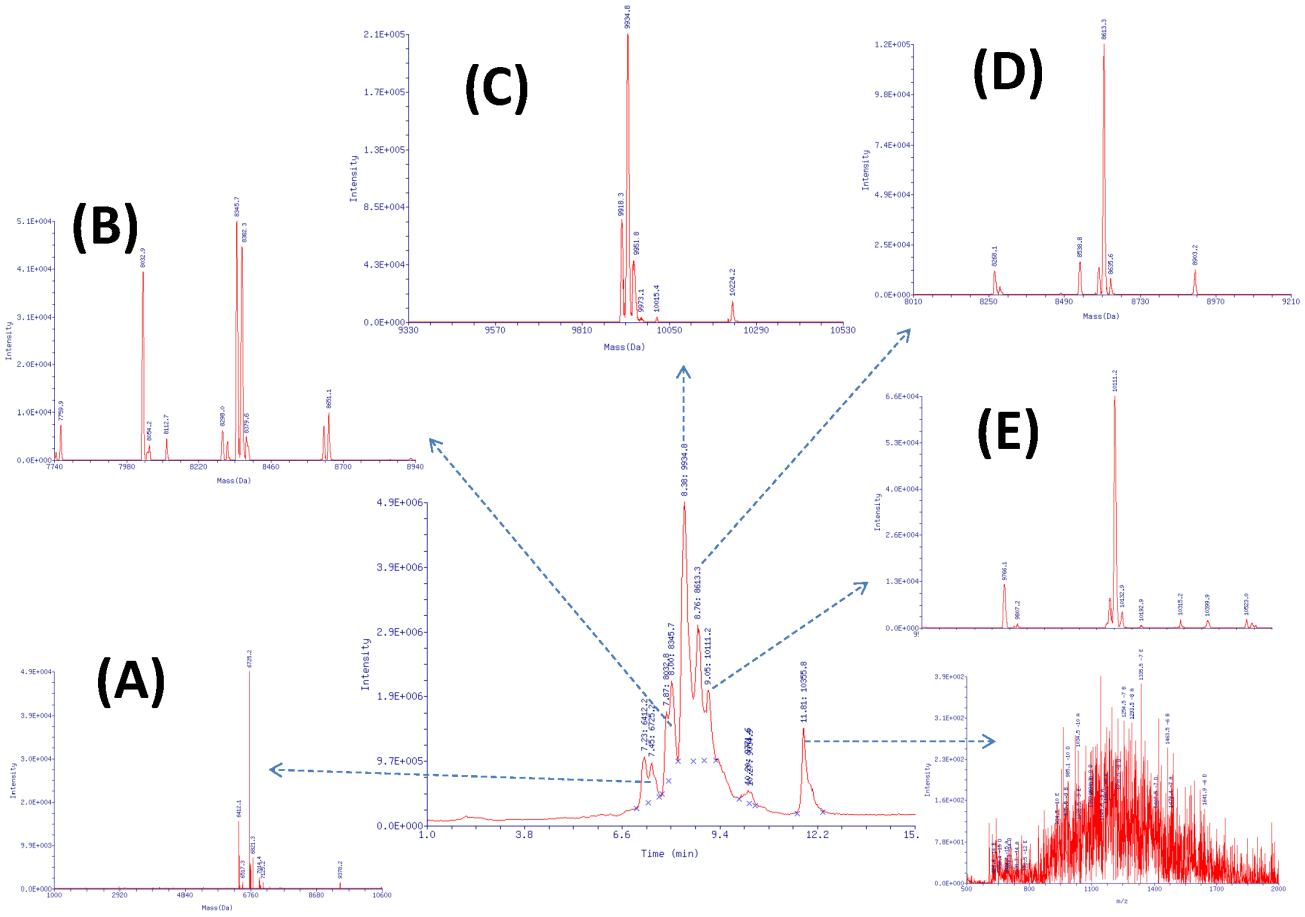
The LC/UV Chromatogram and Deconvoluted Mass Spectrum of the *A*G PEAR product. Components: (A) RT = 7.45 min: MW = 6742.0; (B) RT = 8.00 min; (C) RT = 8.38 min; (D) RT = 8.76 min; (E) RT = 9.05 min; See Table S2 in File S1 for detailed characterization of components.

To obtain full length oligonucleotides, we conducted PEAR reactions in which both A and G were modified (*A*G). As shown in Figure 4 and Table S2 in File S1, in the PspGI digestion of dual modified products, all modified strands are in full length, and the observed MWs are fully consistent with the calculated MW of corresponding expected ODN strands, indicating that the molecular structures of the products are correct. In addition, the total LC/MS area percent of full length modified oligonucleotides reaches up to 100.0% when the residual protein fraction and the unmodified oligonucleotides fraction was omitted, suggesting that PEAR method is well-suited for producing modified oligonucleotides with extremely high purity.

In addition, as shown in Figure S3 in File S1, the sequences of the PEAR products comprise two to dozens of tandem repeats of the target oligonucleotide and PspGI recognition site, which is fully consistent with expectation. Such a high accuracy of the repeat sequences, and the high purity of the product, is guaranteed not only by the use of Phusion high fidelity DNA polymerase, but, more importantly, because of the positive feedback amplification of the target, generation of any non-target sequence is prohibited due to lack of an appropriate template structure.

## Discussion

Based on the “slipping and cleaving mechanism”, PEAR amplifies a specific target oligonucleotide through polymerase elongation and endonuclease cleaving under control of thermal cycling. Compared to traditional *de novo* synthesis, PEAR has several advantages such as lower equipment cost, easier purification process, higher product purity, the ability to avoid failure sequences, and the use of hazardous chemicals. Therefore, PEAR can be a useful tool for large-scale production of modified oligonucleotides, as it allows the production of a large quantity of modified oligonucleotides starting from a small amount of seeds using a simple thermocycler, the only equipment required.

Usually, ASODNs are used in the form of single-stranded [21]–[24], while duplex oligonucleotide, in which the antisense strand is modified with appropriate chemical group, have demonstrated increased cellular uptake, improved potency and in vitro stability when compared to single-stranded ASODNs [25] or small interfering RNA [26]. In addition, the sense and antisense strands of a target ODN could be both useful, since they have been frequently used as controls for each other in biomedical studies. Traditionally, sense and antisense strand of a target ODN were synthesized separately, purified, mixed together and annealed to form double-stranded ODNs if desired. In contrast, using PEAR both strands of an oligonucleotide were produced simultaneously. When a double-stranded ODN is needed, with the high product purity, they can be used directly even without purification. When single strands are required, however, the two strands can be separated from each other by a routine denaturing HPLC [19].

In the present scenario only dATPαS, dGTPαS and dCTPαS, but not dTTPαS, could be incorporated into a desired oligonucleotide. It is, however, often sufficient to resist nuclease degradation by modifying only 3 to 6 bases in the ends of an oligonucleotide. Chimeric ODNs comprises both natural and modified bases are often more favorable for use *in vivo*, since some nascent bases are required for triggering cellular RNase H to recognize and degrade the target RNA transcripts. In addition, in CpG ODNs, one or more unmodified CpG dinucleotides are required to stimulate the innate immune responses.

Thiotriphosphate oligonucleotides, as well as 2′-MOE and LNA modifications, have been widely used in biomedical studies due to their higher potency and *in vivo* stability. It has been reported that LNA ODNs can be produced enzymatically [12]–[14]. Unfortunately, however, although in principle it might be feasible to amplify locked nucleic acid analogues by PEAR, the necessary substrates, LNA modified dNTPs, have been patented but not commercialized, so that their use in enzymatic preparation of oligonucleotides are greatly limited.

## Methods

### Materials

Phusion high fidelity DNA polymerase, highly thermostable restriction enzyme PspGI and dNTPs are purchased from *New England Biolabs*, *Inc*. The recognition site (R) of PspGI is CCWGG, where W = A or T. Synthetic ODNs, including a target (*X*) and a probe (*P*), are synthesized by *Integrated DNA Technologies, Inc.* and purified by HPLC. The sequence of *X* is: TGT AAA CAT CCT CGA CTG GAA G, which is derived from human microRNA hsa-miR-30a. The structure of *P* is *X'R'X'R'X'*, where *X*' and *R'* is complementary respectively to *X* and *R*. The sequence of *P* is: CTT CCA GTC GAG GAT GTT TAC ACC AGG CTT CCA GTC GAG GAT GTT TAC ACC AGG CTT CCA GTC GAG GAT GTT TAC A, where the recognition site of PspGI is underlined. Four 2′-deoxyribonucleotides-5′-O-(1-Thiotriphosphate) (dNTPαSs), including dATPαS, dGTPαS, dCTPαS and dTTPαS were purchased from *Trilink BioTechnologies*, *Inc*.

### PEAR Reactions

PEAR reactions were run in a 96-well Applied Biosystems 9700 Thermal Cycler, each in a 100 µL volume reaction mixture containing 200 µM each dNTP, 15 mM Tris-HCl, 30 mM KCl, 5 mM (NH_4_)_2_SO_4_, 2.5 mM MgCl_2_, 0.02% BSA, 0.08 U/µL Phusion DNA polymerase, 0.4 U/µL PspGI, 0.1 µM target and 1.0 µM probe (or seeds PEAR product). In desired reactions, one or two natural dNTPs were completely replaced with the corresponding dNTPαS (dATPαS, dTTPαS, dCTPαS or dGTPαS). The reactions were initiated at 95°C for 1 min, followed by 35 cycles of denaturing at 95°C for 15 sec, annealing at 55°C for 35 sec, elongation and cleaving at 75°C for 3 to 5 min. If desired, PspGI digestion of the product is conducted under 75°C for 1 to 16 h by adding 0.1 volume 10X NEBuffer 4, 0.4U/µL PspGI, and ddH_2_O to 2X volume. PEAR products were examined by non-denaturing polyacrylamide gel electrophoresis (PAGE) in 15% gels at 5V/cm, stained with ethidium bromide and detected by an UV illuminator. Yields of products were estimated by absorbance measurements OD_260_ of diluted samples.

### Mass Spectrometry Analysis of PspGI Digested PEAR Products

PEAR products were fully digested by the addition of 1 volume of cleavage mixture containing 1X NEBuffer 4, and 1.0 U/µL of PspGI. Cleavage reactions were incubated for 8 hours at 75°C. Before and/or after PspGI digestion, the products were ethanol precipitated, washed twice with 75% ethanol, dried and resuspended in ddH_2_O to remove enzymes, BSA and excessive dNTPs. Electrospray ionization liquid chromatography mass spectrometry (ESI/LC/MS) analysis was performed by *Novatia, LLC* using their High-Throughput Characterization System (HTCS) [20] to characterize the product oligonucleotides and profiling for components.

### Cloning and Sequencing of PEAR Products

TA cloning vector pMD18-T and *E. coli* DH5α competent cells were purchased from *TaKaRa Co. Ltd.*, and operated following the manufacturer’s instructions. To allow efficient TA cloning, PEAR products were pretreated with Taq DNA polymerase in the presence of dNTPs to fill in the sticky ends, and to add an additional adenine nucleotide at the 3′-ends. After ligation, transformation, plating and overnight culture, *E. coli* colonies were picked randomly, plasmids were extracted, double-digested with EcoRI and HindIII and screened using PAGE electrophoresis to identify inserted fragments. Fifty clones containing insertions were sequenced using Sanger method. Mutation rate was computed by dividing the number of mutations by the total number of nucleotides.

## Supporting Information

File S1 **Figure S1,** **PAGE electrophoresis of PspGI digestions of PEAR products.** (A) Digestion of natural PEAR products;(B) Digestion of PEAR products incorporating PS-dATP; (C) Digestion of PEAR products incorporating PS-dGTP. **Figure S2, The nucleotide sequence of the recognition site of PspGI. Figure S3, Sanger sequencing of PEAR products.** (Target sequence repeats are underlined in blue; PspGI restriction enzyme recognition sites are underlined in yellow.) **Figure S4,** The LC/UV Chromatogram and Deconvoluted Mass Spectrum of the *A*G PEAR product incorporating dATPαS+ dGTPαS fully digested by PspGI. Components: (A) RT = 7.45 min: MW = 6742.0; (B) RT = 8.00 min; (C) RT = 8.38 min; (D) RT = 8.76 min; (E) RT = 9.05 min; See Table S2 for detailed characterization of components. **Table S1,** Characterization components of the *A PEAR product by LC/UV/MS analysis. Table S2, Characterization components of the *A*G PEAR product by LC/UV/MS analysis.
(DOC)
