# Supplementary material for: Preparation of 5′-O-(1-Thiotriphosphate)-Modified Oligonucleotides Using Polymerase-Endonuclease Amplification Reaction (PEAR)
Source: PLoS One. 2013 Jul 4;8(7):e67558. doi: 10.1371/journal.pone.0067558 (PMC3701678; doi:10.1371/journal.pone.0067558)
Supplement: File S1 — Figure S1, PAGE electrophoresis of PspGI digestions of PEAR products. (A) Digestion of natural PEAR products;(B) Digestion of PEAR products incorporating PS-dATP; (C) Digestion of PEAR products incorporating PS-dGTP. Figure S2, The nucleotide sequence of the recognition site of PspGI. Figure S3, Sanger sequencing of PEAR products. (Target sequence repeats are underlined in blue; PspGI restriction enzyme recognition sites are underlined in yellow.) Figure S4, The LC/UV Chromatogram and Deconvoluted Mass Spectrum of the *A*G PEAR product incorporating dATPαS+ dGTPαS fully digested by PspGI. Components: (A) RT = 7.45 min: MW = 6742.0; (B) RT = 8.00 min; (C) RT = 8.38 min; (D) RT = 8.76 min; (E) RT = 9.05 min; See Table S2 for detailed characterization of components. Table S1, Characterization components of the *A PEAR product by LC/UV/MS analysis. Table S2, Characterization components of the *A*G PEAR product by LC/UV/MS analysis. (DOC) [file pone.0067558.s001.doc]

**Supporting Information for:**

**Preparation of 5'-O-(1-Thiotriphosphate)-modified Oligonucleotides Using Polymerase-endonuclease Amplification Reaction (PEAR)**

Biao Li^1#^, Shihua Dong^1#^, Jiajun Wu^1^, Jianye Zhang^1^, Gang Chen^1^, Quanjiang Dong^2^, Xinhong Zhu^2^ and Xiaolong Wang^1*^

1. Department of Biotechnology, Ocean University of China, Qingdao, 266003, People's Republic of China

Qingdao Municipal Hospital, Qingdao 266021, People's Republic of China

**E-mail:* [*Xiaolong@ouc.edu.cn*](mailto:Xiaolong@ouc.edu.cn)

1. **Materials & Methods**
   1. **Materials**

Phusion high fidelity DNA polymerase, highly thermostable restriction enzyme PspGI and dNTPs are purchased from *New England Biolabs*, *Inc*. The recognition site (R) of PspGI is CCWGG, where W=A or T. Synthetic ODNs, including a target (*X*) and a probe (*P*), are synthesized by *Integrated DNA Technologies, Inc.* and purified by HPLC. The sequence of *X* is: TGT AAA CAT CCT CGA CTG GAA G, which is derived from human microRNA hsa-miR-30a. The structure of *P* is *X'R'X'R'X'*, where *X*' and *R'* is complementary respectively to *X* and *R*. The sequence of *P* is: CTT CCA GTC GAG GAT GTT TAC ACC AGG CTT CCA GTC GAG GAT GTT TAC ACC AGG CTT CCA GTC GAG GAT GTT TAC A, where the recognition site of PspGI is underlined. Four 2'-deoxyribonucleotides-5'-O-(1-Thiotriphosphate) (dNTPαSs), including dATPαS, dGTPαS, dCTPαS and dTTPαS were purchased from *Trilink BioTechnologies*, *Inc*. Their molecular structure representations are shown in Figure 1.

- 1. **PEAR reactions**

PEAR reactions were run in a 96-well Applied Biosystems 9700 Thermal Cycler, each in a 100 µL volume reaction mixture containing 200 µM each dNTP, 15 mM Tris-HCl, 30 mM KCl, 5 mM (NH_4_)_2_SO_4_, 2.5 mM MgCl_2_, 0.02 % BSA, 0.08 U/µL Phusion DNA polymerase, 0.4 U/µL PspGI, desired amount of seeds (target and probe). In desired reactions, one or two natural dNTPs were completely replaced with the corresponding dNTPαS (dATPαS, dTTPαS, dCTPαS or dGTPαS). The reactions were initiated at 95℃ for 1 min, followed by 35 cycles of denaturing at 95℃ for 15 sec, annealing at 55℃ for 35 sec, elongation and cleaving at 75℃ for 3 to 5 min. If desired, PspGI digestion of the product is conducted under 75℃ for 1 to 16h by adding 0.1 volume 10X NEBuffer 4, 0.4U/µL PspGI, and ddH_2_O to 2X volume. PEAR products were examined by non-denaturing polyacrylamide gel electrophoresis (PAGE) in 15% gels at 5V/cm, stained with ethidium bromide and detected by an UV illuminator. Yields of products were estimated by absorbance measurements OD_260_ of diluted samples.

- 1. **Mass spectrometry analysis of PEAR Products**

PEAR products were fully digested by the addition of 1 volume of cleavage mixture containing 1X NEBuffer 4, and 1.0 U/µL of PspGI. Cleavage reactions were incubated for 8 hours at 75℃. Before and/or after PspGI digestion, the products were ethanol precipitated, washed twice with 75% ethanol, dried and resuspended in ddH_2_O to remove enzymes, BSA and excessive dNTPs. Electrospray ionization liquid chromatography mass spectrometry (ESI/LC/MS) analysis was performed by *Novatia, LLC* using their High-Throughput Characterization System (HTCS) [*19*] to characterize the product oligonucleotides and profiling for components.

- 1. **Cloning and Sequencing of PEAR Products**

TA cloning vector pMD18-T and *E. coli* DH5α competent cells were purchased from *TaKaRa Co. Ltd.*, and operated following the manufacturer’s instructions. To allow efficient TA cloning, PEAR products were pretreated with Taq DNA polymerase in the presence of dNTPs to fill in the sticky ends, and to add an additional adenine nucleotide at the 3’-ends. After ligation, transformation, plating and overnight culture, *E. coli* colonies were picked randomly, plasmids were extracted, double-digested with EcoRI and HindIII and screened using PAGE electrophoresis to identify inserted fragments. Fifty clones containing insertions were sequenced using Sanger method. Inserted sequences were aligned with ClustalW2. Mutation rate was computed by dividing the number of mutations by the total number of nucleotides.

1. **RESULTS**
   1. **Digestion of modified PEAR products**

During each cycle of PEAR, the number of product molecules are increasingly excessive than that of PspGI, so that the products were not fully digested. After PEAR amplification, products were digested by adding sufficient PspGI and incubating at 75℃ for a longer duration if a complete digestion is desired. As shown in Figure S1A, when a natural PEAR product was digested by PspGI, a lowest 27-bp band representing the monomeric target ODNs was produced. The upper bands represent multiple-repeat species that were resulted from partial digestions. With increasing digestion time, these multiple repeats were dissociated gradually into monomers. As shown in Figure S1B, PspGI digestion of a PEAR product incorporating dATPαS is basically same to that of the natural product, indicating that insertion of a modified base (dATPαS) in the middle of the PspGI recognition site (CCWGG) has no obvious influence on this enzyme in recognizing and cutting the products.

**
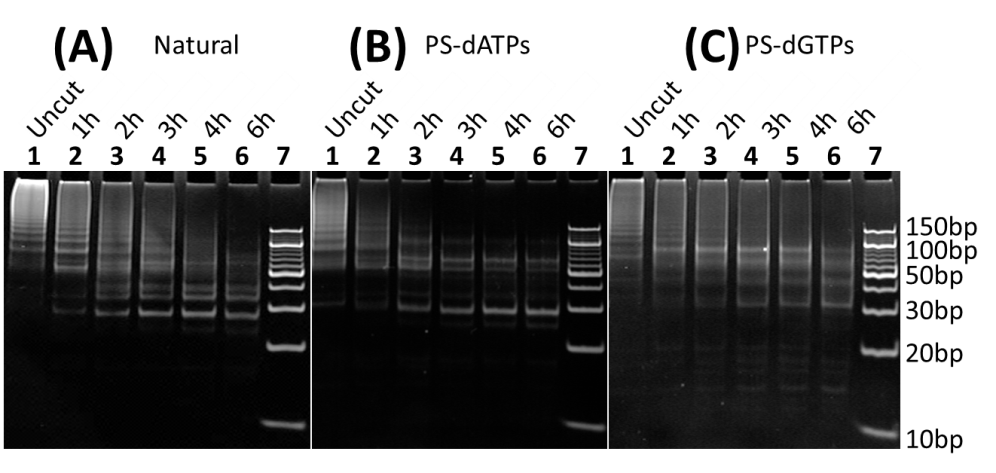
**

Figure S1. PAGE electrophoresis of PspGI digestions of PEAR products.

(A) Digestion of natural PEAR products;(B) Digestion of PEAR products incorporating PS-dATP; (C) Digestion of PEAR products incorporating PS-dGTP;

However, as shown in Figure S1C, modified PEAR product incorporating dGTPαS are more resistant to PspGI digestion than the natural products. As shown in Figure S1D, the natural product and the PEAR products incorporating dATPαS or dCTPαS were fully digested by PspGI in 4 or 8h, the product incorporating dGTPαS, however, were not fully digested in 8h, but in16h. In addition, as shown in Figure S2A, the lengths of the PEAR products incorporating dCTPαS (lane 11) are shorter than that of other PEAR products, suggesting that PEAR products incorporating dCTPαS are more sensitive to PspGI digestion than their natural counterparts. Considering the recognition site of PspGI (Figure S2), it is suggested that the endonucleolytic activity of PspGI is reduced by modified Gs (shown in bold) located in the opposite strand, but enhanced by modified Cs (shown in italic) located in the same strand, of the cutting site. This is surprisingly unexpected because it has been generally believed that thiotriphosphate modifications always enhance the resistance of nucleic acids against nuclease attack.


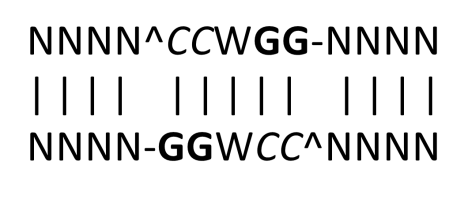


Figure S2. The nucleotide sequence of the recognition site of PspGI.

- 1. **Cloning and sequencing of the PEAR product**

To test the accuracy of the sequence of the PEAR products, we cloned a natural PEAR product into *E*. *coli* DH5α using pMD18-T vector. Fifty clones were screened and sequenced by Sanger method. As shown in Figure S3, the sequences of the products comprise two to dozens of tandem repeats of the target oligonucleotide and PspGI recognition site, which is fully consistent with our expectation. Mutation rate analysis suggested that the accuracy of the repeat sequences reaches >99.9999%.


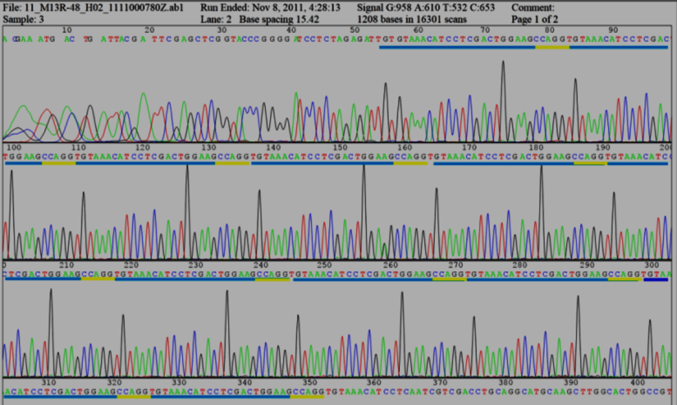


Figure S3 Sanger sequencing of PEAR products (Target sequence repeats are underlined in blue; PspGI restriction enzyme recognition sites are underlined in yellow).

- 1. **LC/MS profiling of PEAR amplified oligonucleotides**

We employed LC/MS analysis to measure the molecular weight (MW), confirm the molecular structure and profile the components of the PEAR products. As shown in Figure 3 and Table S1, in the digestion of the PEAR product with modified As (A*), there are five DNA strands, including a pair of unmodified strands which are the original target and probe, and three modified strands that were produced by PEAR amplification. Among the five strands, A, B and C are all in full length, while the other two ones, D and E, were truncated by one or two 3'-terminal bases. The calculated MW of full length D is 8501.4, but the observed MW is only 8173.6, indicating that it is a truncation of D by the 3'-terminal nucleotide G (calculated MW 8172.18). Moreover, the calculated MW of full length E is 10067.44, but the observed MW is only 9410.2, indicating that it is a truncation of E by two 3'-terminal nucleotides GG (calculated MW 9409.0).

To get full length products, we prepared oligonucleotides in which both A and G bases were modified (A*G*). As shown in Figure S4 and Table S2, in the PspGI digestion of double modified products, all DNA strands are in full length, and the observed MWs are consistent with the calculated MW of corresponding expected DNA strands, indicating that the molecular structures of the products are correct. In addition, as shown in Figure S4 and Table S2, the last three peaks (RT=10.20, 10.27 and 11.81, area percent=1.11, 0.73 and 11.34) were characterized as residual enzymes. So the area percent of full length modified oligonucleotides was considered as 100.0% when the fractions of proteins and initial target and probe fractions were omitted.


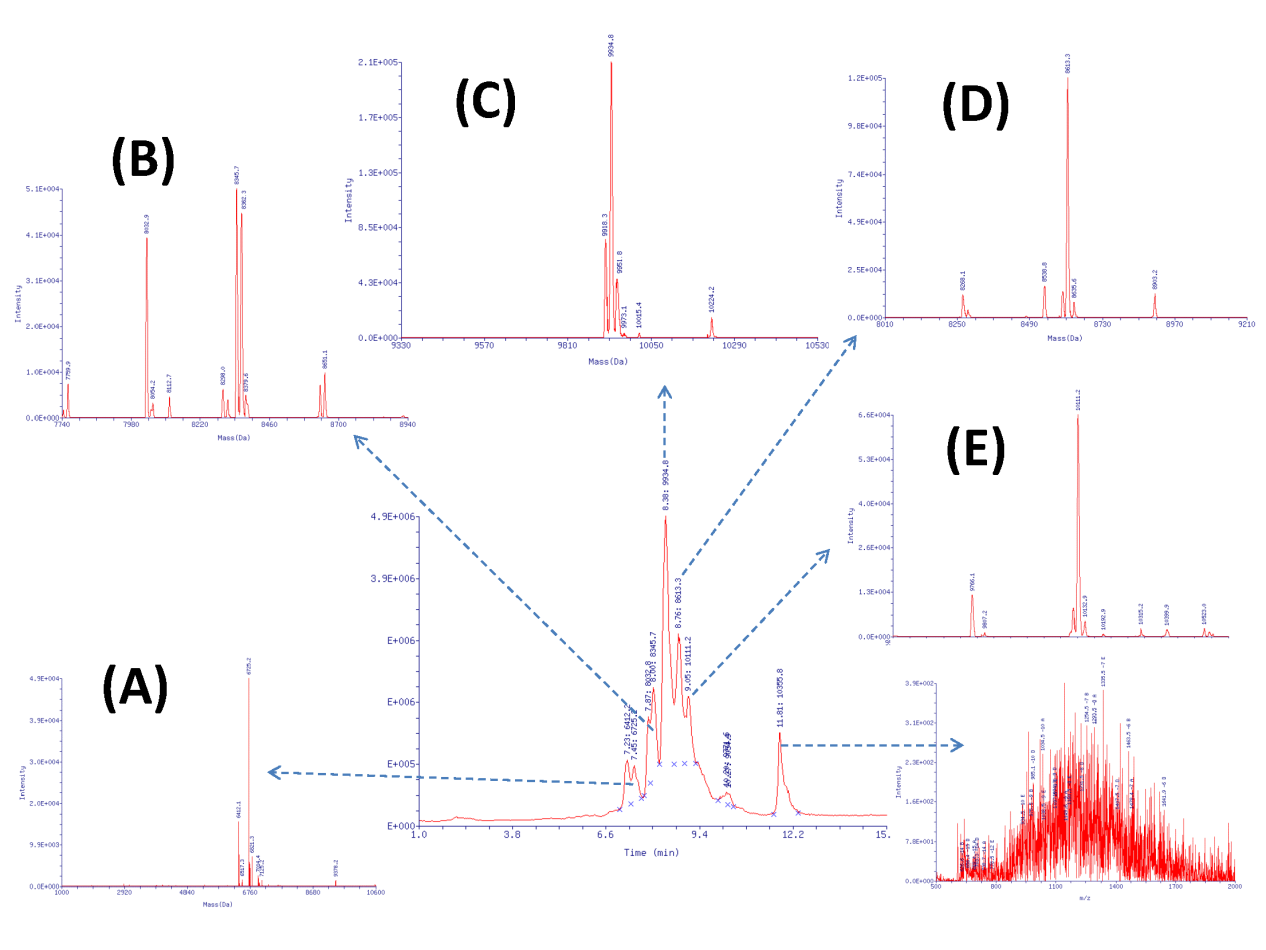


**Figure S4.** The LC/UV Chromatogram and Deconvoluted Mass Spectrum of the *A*G PEAR product incorporating dATPαS+ dGTPαS fully digested by PspGI; Components: (A) RT = 7.45 min: MW=6742.0; (B) RT = 8.00 min; (C) RT = 8.38 min; (D) RT = 8.76 min; (E) RT = 9.05 min; See Table S2 for detailed characterization of components.

Table S1. Characterization components of the *A PEAR product by LC/UV/MS analysis

| Retention  Time  (Min) | Component | Strand | Modification  Status | Structure  Representation | Calculated  MW  (Da) | Observed  MW  (Da) | LC/MS  Area  percent | **Sequence**  **(* thiophosphate diester bond, underlined: PspGI recognition site)** |
| --- | --- | --- | --- | --- | --- | --- | --- | --- |
| 7.35 | Target (A) | Sense | Unmodified | X | 6743.4 | 6742.0 (Full Length) | 7.64 | 5’-TGTAAACATCCTCGACTGGAAG-3’ |
| 7.90 | Probe (B) | Antisense | Unmodified | X’-R’ | 8362.4 | 8363.2 (Full Length) | 36.45 | 3’-ACATTTGTAGGAGCTGACCTTCGGTCC-5’ |
| 8.23 | Product (C) | Antisense | Modified | (X’-R’)^*A^ | 8426.4 | 8427.7 (Full Length) | 17.74 | 3’-*AC*ATTTGT*AG*GAGCT*GACCTTCGGTCC-5’ |
| 8.46 | Product (D) | Sense | Modified | (R-X)^*A-G^ | 8172.18 | 8173.6 (Minus 3’-G) ^a^ | 33.30 | 5’-CC*AGGTGT*A*A*AC*ATCCTC*GACTGG*A*A~~G~~-3’ |
| 8.66 | Product (E) | Sense | Modified | (R-X-R)^*A-GG^ | 9409.0 | 9410.2 (Minus 3’-GG) ^b^ | 4.87 | 5’-CC*AGGTGT*A*A*AC*ATCCTC*GACTGG*A*AGCC*A~~GG~~-3’ |
| Total |  |  | |  | |  | 100.0 |  |

**Note**:

1. The calculated MW of full length (R-X)^*A^ is 8501.4, but the observed MW is only 8173.6, indicating that it is a truncation of (R-X)^*A^ by the 3'-terminal nucleotide G (calculated MW 8172.18).
2. The calculated MW of full length (R-X-R)^*A^ is 10067.44, but the observed MW is only 9410.2, indicating that it is a truncation of E by two 3'-terminal nucleotides GG (calculated MW 9409.0).

Table S2. Characterization components of the *A*G PEAR product by LC/UV/MS analysis

| **Retention**  **Time (Min)** | **Component** | **Strand** | **Modification**  **Status** | **LCMS Area**  **percent** | **Structure** | **Calculated**  **MW (Da)** | **Observed**  **MW (Da)** | **Sequence**  **(* thiophosphate diester bond, underlined: PspGI recognition site)** |
| --- | --- | --- | --- | --- | --- | --- | --- | --- |
|  | **Target** | Sense | Unmodified | 0 | **X** | 6743.40 | Not observed ^a^ | 5’-TGTAAACATCCTCGACTGGAAG-3’ |
| 7.45 | **Probe (A)** | Antisense | Unmodified | 5.21 | **X’** | 6412.18 | 6412.2 | 3’-~~A~~CATTTGTAGGAGCTGACCTTC-5’ |
|  |  |  |  | 4.37 | **X’ ^-A^** | 6725.40 | 6725.2 (Full Length) | 3’-ACATTTGTAGGAGCTGACCTTC-5’ |
| 8.00 | **Probe (B)** | Antisense | Unmodified | 4.47 | **X’-R’^-A^** | 8033.25 | 8032.8 (Minus 3’-A) | 3’-~~A~~CATTTGTAGGAGCTGACCTTCGGTCC-5’ |
|  |  |  |  | 9.68 | **X’-R’** | 8346.47 | 8345.7 (Full Length) | ACATTTGTAGGAGCTGACCTTCGGTCC |
|  |  |  |  |  | **X’-R’^*G^** | 8362.47 | 8362.3 (Full Length) | ACATTTGTAGGAGCTGACCTTC G*GTCC |
|  |  |  |  |  | **X’-R’^*G*G^** | 8378.47 | 8379.6 (Full Length) | ACATTTGTAGGAGCTGACCTTC*G*GTCC |
| 8.38 | **Product (C)** | Antisense | Modified | 37.09 | **R’^*A*G^-X’-R’** | 9919.54 | 9918.3 (Full Length) | *G*GTCCACATTTGTAGGAGCTGACCTTCGGTCC |
|  |  |  |  |  | **R’-X’-R’^*A*G^** | 9919.54 | 9918.3 (Full Length) | GGTCCACATTTGTAGGAGCTGACCTTC*G*GTCC |
|  |  |  |  |  | **R’^*A*G^-X’-R’^*A*G^** | 9935.54 | 9934.8 (Full Length) | *G*GTCCACATTTGTAGGAGCTGACCTTCG*GTCC |
|  |  |  |  |  | **R’^*A*G^-X’-R’^*A*G^** | 9951.54 | 9951.8 (Full Length) | *G*GTCCACATTTGTAGGAGCTGACCTTC*G*GTCC |
| 8.76 | **Product (D)** | Sense | Modified | 17.41 | **(X-R)^*A*G^** | 8613.50 | 8613.3 (Full Length) | T*GT*A*A*AC*ATCCTC*G*ACT*G*G*A*A*GCC*A*G*G |
| 9.05 | **Product (E)** | Antisense | Modified | 8.58 | **(R’-X’-R’) ^*A*G^** | 10111.54 | 10111.2 (Full Length) | *G*GTCC*AC*ATTT*GT*A*G*G*A*GCT*G*ACCTTC*G*GTCC |
| 10.2 | **Enzyme** |  |  | 1.11 |  |  |  |  |
| 10.27 | **Enzyme** |  |  | 0.73 |  |  |  |  |
| 11.81 | **Enzyme** |  |  | 11.34 |  |  |  |  |
|  | **Total** |  |  | 99.99 |  |  |  |  |

**Note**: (a) The unmodified sense strand (the initial targets) might be completely digested by the exonuclease activity of the Phusion DNA polymerase during PEAR amplification or after PspGI digestion, since the initial concentration of the target sense strand was 10 times lower than that of the antisense strand.
